# Development of a heating reactor for a continuous flow-through application in urea measurement

**DOI:** 10.1155/S1463924603000221

**Published:** 2003

**Authors:** S. S. Randhawa, A. K. Ganju, R. P. Bajpai

**Affiliations:** Central Scientific Instruments Organisation Chandigarh 160030 India

## Abstract

In most biochemical analyses, a flow-through heating arrangement is needed to reduce the reaction time or maintain a constant temperature. A rectangular reactor is described that is constructed of aluminium, is hollow inside and is filled with silicone oil. The glass coil through which the solution flows is immersed in the silicone oil. The heater, a Peltier-effect heat pump, on one side and the temperature sensor on the other side of the reactor body are embedded for heating and temperature control. The brief performance evaluation of the reactor is discussed by measuring the absorbance of urea concentration at different temperatures.


Journal of Automated Methods & Management in Chemistry
Vol. 25, No. 6 (November–December 2003) pp. 129–132

Taylor & Francis

Taylor & Francis Group

### Development of a heating reactor for a continuous flow-through application in urea measurement

**S. S. Randhawa*, A. K. Ganju and R. P. Bajpai**
*Central Scientific Instruments Organisation, Chandigarh — 160030, India*

*In most biochemical analyses, a flow-through heating arrangement is needed to reduce the reaction time or maintain a constant temperature. A rectangular reactor is described that is constructed of aluminium, is hollow inside and is filled with silicone oil. The glass coil through which the solution flows is immersed in the silicone oil. The heater, a Peltier-effect heat pump, on one side and the temperature sensor on the other side of the reactor body are embedded for heating and temperature control. The brief performance evaluation of the reactor is discussed by measuring the absorbance of urea concentration at different temperatures.*

### Introduction

In flow analysis, a flow-through heating arrangement is necessary to accomplish a desired reaction within a reasonable time. The use of in-line microwave heating/digestion has gained in popularity in recent years because of its ability to perform contactless energy heating. However, this approach is not practical with field instruments [1]. Regarding the use of conventional heated reactors, they have been in use over the past decade for measurement of formaldehyde (HCHO) [2], carbonyl compounds in general [3], ammonia [4] and peroxides [5], for the field measurement of atmospheric formaldehyde [6], and for water borne arsenic [7]. Heated reactors based on polymeric tubing are most common and such reactors are typically used in a manner in which they are put in a heated bath or an otherwise thermally conductive potting in which a heater and a temperature sensor are embedded for heating and temperature control. In more recent examples, to improve heat transfer and thus energy efficiency, some workers have used polymeric tubing, which is woven on a stainless steel screen in a serpentine design [8], potted in a low melting bismuth alloy along with a platinum resistance temperature detector sensor and a pair of silicone-embedded heaters [9].

The present paper describes a heating reactor that can be used in continuous flow analysis for the determination of biochemical parameters in clinical biochemistry, and food and environmental chemistry.

* To whom correspondence should be addressed.
e-mail: ss_randhawa1@rediffmail.com

### Experimental

- 2.5% diacetyl monoxime.
- 0.5% thiosemicabazide.
- Working urea colour reagent: 20 ml 2.5% diacetyl monoxime and 20 ml 0.5% thiosemicarbazide, transferred to a 100-ml volumetric flask, made to 100 ml with distilled water and shaken thoroughly.
- Ferric chloride-phosphoric acid: dissolved 15 g ferric chloride in about 30 ml water and transferred to a 500-ml glass stopper graduated mixing cylinder. A total of 300 ml 85% phosphoric acid is slowly added. It is diluted to 450 ml with water and mixed.
- 15% Sulphuric acid.
- Working urea acid: 20 ml ferric chloride-phosphoric acid is placed into a 500-ml volumetric flask and diluted to volume with 15% sulphuric acid.
- Standard urea solution: 200 mg% solution is prepared in distilled water.

*Heating system*

The heating reactor (figure 1) consists of an aluminium body, heater, temperature sensor, glass coil and silicone oil. The heater, Peltier-effect heat pumps (two numbers), are used at 15.4 V and function at 33.4 W. It raises the temperature of the housing and heats the oil in the tank. It also heats the sample moving through the heating coil. The LM35DZ is a three-terminal integrated circuit plastic packaged temperature sensor with a linear voltage output of 10 mV/°C. It is ideally suited for ambient temperature measurement from 0 to 100°C and its operating voltage range is +30 to +4 V. Silicone oil is used to maintain the heating element at a constant temperature. The heating coil is made of glass and fixed on a thermometer housing, which is immersed in the coil chamber. The internal diameter of this glass tube is 1.6 mm and the wall thickness is 1 mm.

The simple circuit of figure 2 provides good temperature control. In series with the heater DS1, the relay K1 is used. To the collector of SL 100, driving transistor for current requirement, relay-exciting coil is connected. The temperature sensor LM35DZ is used for temperature sensing. The output of the sensor in terms of millivolts is directly proportional to the change in temperature. The regulator IC7805 supplies a constant 5 V to the sensor. One of the inputs of the operational amplifier 741 is from LM35DZ at the non-inverting terminal. Another input to the operational amplifier is from the variable pot R5, which serves the reference input in millivolts depending on the desired temperature. A +12 V DC supply for the relay coil was obtained by a step-down transformer and a bridge rectifier. The output of the rectifier is given to regulator IC7812

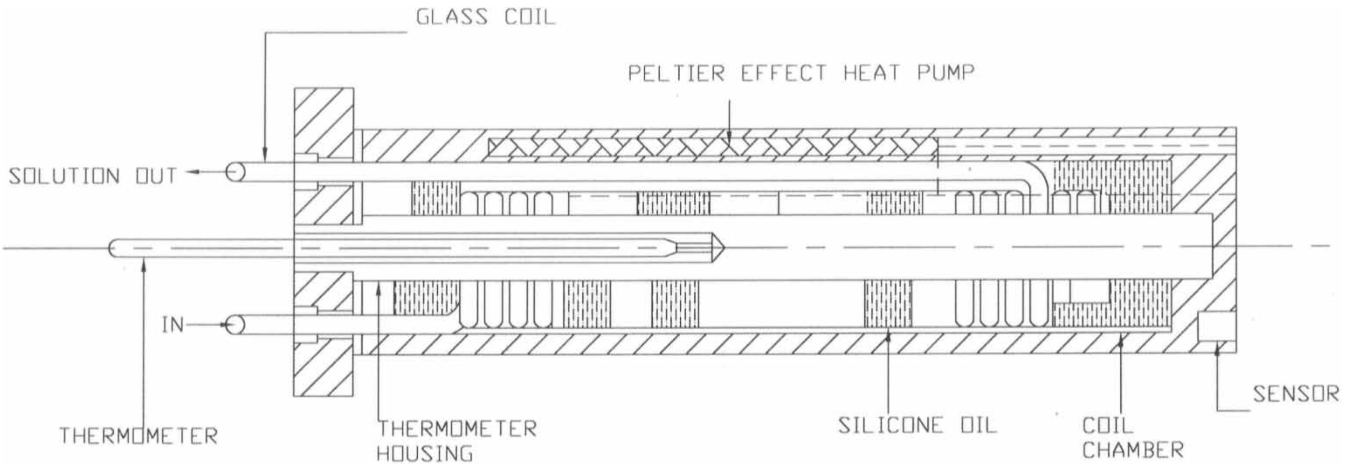

*Figure 1. Schematic diagram of heating reactor.*

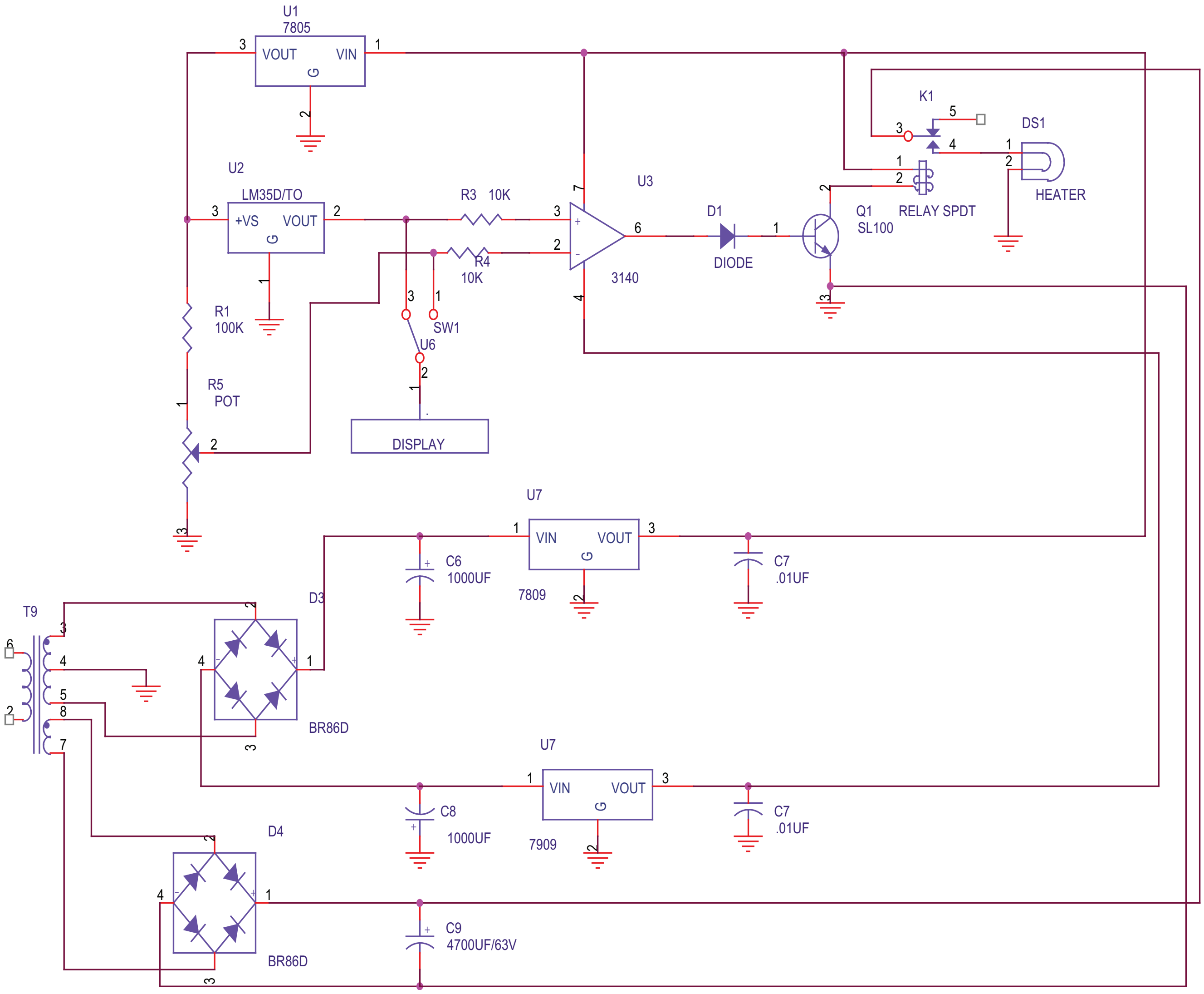

*Figure 2. Circuit diagram of temperature controller.*

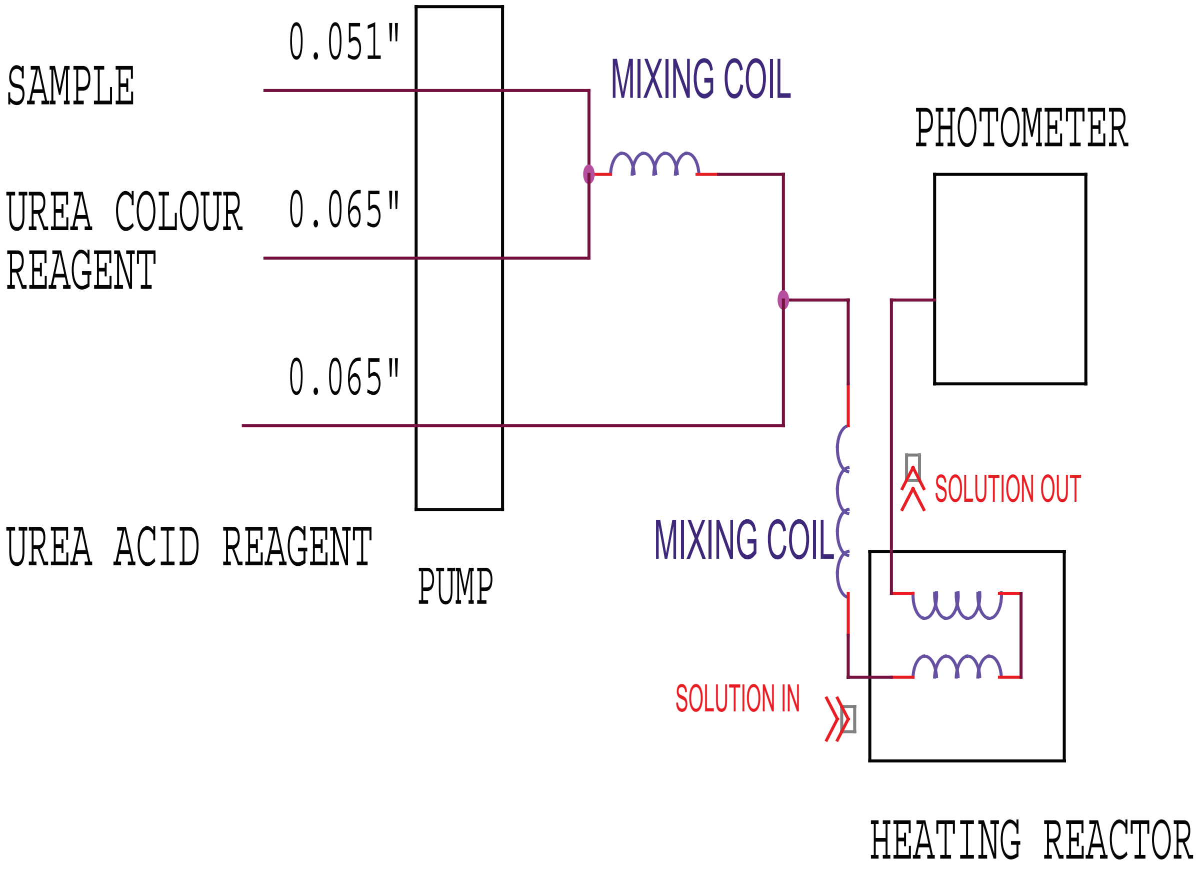

*Figure 3. Flow diagram of urea measurement.*

that supplies a constant 12 V to the relay coil. As per the reference set by R5, the operational amplifier sets its output to switch either on or off relay so as to maintain a constant temperature in the chamber.

The development of the pump assembly used for pumping the solution is discussed in the authors' earlier work [10]. The improved photometric system has a tungsten lamp as a light source, a photo diode as a detector and a microcontroller 8051 is used for processing and displaying absorbance [10]. The interference filter composed of an externally thin semireflecting metallic film of wavelength 520 nm is used. A stream of urea colour reagent joins the urea stream. These two solutions of urea and colour reagent are mixed by a mixing glass coil. The other stream of urea acid reagent is joined as shown in figure 3. After mixing these solutions, it is passed through the heating reactor at a particular temperature. As the acidified solution passes through the heating reactor, urea reacts with diacetyl in the presence of ferric ions. The pink colour of the reaction product is intensified by the presence of thiosemicarbazide. The colour reaction product is measured in a 10-mm flow cell at 520 nm [11].

### Results and discussion

In 1834, Jean C. A. Peltier discovered that the passage of an electric current through a junction of two dissimilar conductors can either cool or heat the junction depending on the direction of current. Heat generation or absorption rates are proportional to the magnitude of the current and also to the temperature of the junction. Peltier-effect heat pumps consist of many such couples connected electrically in series and thermally in parallel. Semiconductors doped of both the p and n types form the element of the couple and are soldered to copper connecting stripes. Ceramic faceplates electrically insulate these connecting strips from external surfaces. At open circuit, a temperature gradient maintained across the device creates a potential across its terminals which is proportional to the temperature difference. If the temperature difference is maintained and if the device is connected to an electrical load, power is generated. If the device is instead connected to a DC source, heat will be absorbed at one end of the device, cooling it, while heat is rejected at the other end, where the temperature rises. Reversing the current reverses the flow of heat. Therefore, the module can generate electric power or, depending on how it is connected to external circuitry, heat or cool an object. There are many methods of temperature controlling Peltier devices in either open or closed loop modes. With open loop arrangements, manual adjustment of the input current is made, normally by means of a variable power supply, and the temperature is thereby maintained reasonably near the desired set point. With the closed loop method, a temperature sensor is used to sense the temperature of the device and appropriate electronic circuitry effects automatic control of the Peltier device current. Very

*Table 1. Variation in absorbance of standard urea (200 mg/100 ml) concentration heated in the reactor at different temperatures.*

| Sr. no. | Temperature set (°C) | Mean absorbance (AU) ($n = 5$) | Difference in mean absorbance per 10°C |
|---|---|---|---|
| 1 | 32 | 0.051 | – |
| 2 | 42 | 0.135 | 0.084 |
| 3 | 52 | 0.195 | 0.060 |
| 4 | 62 | 0.235 | 0.040 |

precise control can be achieved by this method. The Peltier-effect heat pump module was purchased from RS Components and Controls (India) Ltd., Chandigarh-160020.

Table 1 shows the variation of the absorbance of urea concentration standard solution measured at different temperatures. When the temperature is set at 32°C, the mean absorbance of the urea standard concentration is 0.051 AU (absorbance units). However, as the temperature is increased by 10°C, i.e. fixed at 42°C, then the mean absorbance is 0.135 AU. There is a change of 0.084 AU for 10°C. The mean differences in absorbance at 52 and 62°C are 0.06 and 0.04, respectively. This shows that when the temperature is increased, the absorbance also increases. That means there is heat generation to heat the solution in the system. The absorbance of a standard urea solution is not measured beyond 70°C as the system temperature cannot be increased beyond this temperature with these Peltier-effect heat pump modules. If the temperature is to increase beyond this temperature, then the Peltier-effect heat pumps of higher wattage can be used and their size will also be different.

### Conclusion

The flow type reactor is very low cost in comparison with other existing alternative reactors and is easy to construct. It can also be used practically with micro-controller chips.

### Acknowledgement

The authors thank the Technical Mission on Oil Seeds, Pulses and Maize (TMOP&M), Ministry of Agriculture, Government of India, New Delhi, for funding the project.

### References

1. Caballo Lopez, A. and de Castro, L., *Talanta*, **59** (2003), 837.
2. Fan, Q. and Dasgupta, P. K., *Anal. Chem.*, **66** (1994), 551.
3. Dasgupta, P. K., Zhang, G., Schueze, S. and Marx, J. N., *Anal. Chem.*, **66** (1994), 1965.
4. Genfa, Z., Uehara, T., Dasgupta, P. K., Cearke, T. and Winiwarter, W., *Anal. Chem.*, **70** (1998), 3656.
5. Li, J. and Dasgupta, P. K., *Anal. Chem.*, **72** (2000), 5338.
6. Li, J., Dasgupta, P. K., Genfa, Z. and Hutterli, M. A., *Field Anal. Chem. Technol.*, **5** (2001), 2.
7. Dasgupta, P. K., Huang, H., Zhang, G. and Cobb, G. P., *Talanta*, **58** (2002), 153.
8. Waiz, S., Cedillo, B. M., Jambunatham, S., Hohnholt, S. G., Dasgupta, P. K. and Wolcott, D. K., *Anal. Chem. Acta*, **428** (2001), 163.
9. Dasgupta, P. K., Loree, E. H., Li, J. and Genfa, Z., *Anal. Chem.*, **75** (2003), 3924.
10. Randhawa, S. S., Baban, K. S Bansod, Anirudh K. Singh, Chand, G. and Ganju, A. K., *J. Autom. Meth. Manag. Chem.*, **25** (2003), 51.
11. Marsh, W. H., Fingerhut, B. and Miller, H., *Clin. Chem.*, **11** (1965), 624.

